# Adherence to Traditional Chinese Postpartum Practices and Postpartum Depression: A Cross-Sectional Study in Hunan, China

**DOI:** 10.3389/fpsyt.2021.649972

**Published:** 2021-07-27

**Authors:** Pengfei Guo, Dong Xu, Zeyan Liew, Hua He, Peter Brocklehurst, Beck Taylor, Chao Zhang, Xin Jin, Wenjie Gong

**Affiliations:** ^1^School of Public Health, Sun Yat-sen University, Guangzhou, China; ^2^Department of Environmental Health Sciences, Yale School of Public Health, New Haven, CT, United States; ^3^Yale Center for Perinatal, Pediatric, and Environmental Epidemiology, Yale School of Public Health, New Haven, CT, United States; ^4^School of Health Management, Southern Medical University, Guangzhou, China; ^5^Center for WHO Studies, Southern Medical University, Guangzhou, China; ^6^Institute for Health Management, Southern Medical University, Guangzhou, China; ^7^ACACIA Labs, Institute for Global Health and Dermatology Hospital, Southern Medical University, Guangzhou, China; ^8^Department of Epidemiology, Tulane University School of Public Health and Tropical Medicine, New Orleans, LA, United States; ^9^Birmingham Clinical Trials Unit, University of Birmingham, Birmingham, United Kingdom; ^10^Institute for Applied Health Research, University of Birmingham, Birmingham, United Kingdom; ^11^Xiangya School of Public Health, Central South University, Changsha, China; ^12^Department of Psychiatry, University of Rochester, Rochester, NY, United States

**Keywords:** postpartum practices, doing-the-month, postpartum depression, EPDS, China

## Abstract

**Background:** The relationship between adherence to traditional Chinese postpartum practices (known as “doing-the-month”) and postpartum depression (PPD) remains unknown. Practices including restrictions on diet, housework and social activity, personal hygiene, and cold contact, could introduce biological, psychological, and socio-environmental changes during postpartum.

**Methods:** The cross-sectional study included 955 postpartum women in obstetric clinics in Hunan Province of China between September 2018 to June 2019. Thirty postpartum practices were collected by a self-report online structured questionnaire. Postpartum depression symptoms were assessed by the Chinese version of the Edinburgh Postnatal Depression Scale (EPDS). Multivariable linear regression was used to estimate the differences in EPDS scores according to adherence to postpartum practices. Firth's bias-reduced logistic regression was employed to analyze the binary classification of having PPD symptoms (EPDS ≥ 10).

**Results:** Overall, both moderate and low adherence to postpartum practices appeared to be associated with higher EPDS scores (adjusted difference 1.07, 95% CI 0.20, 1.94 for overall moderate adherence; and adjusted difference 1.72, 95% CI 0.84, 2.60 for overall low adherence). In analyses by practice domain, low adherence to housework-related and social activity restrictions was associated with having PPD symptoms compared with high adherence (OR 1.61, 95% CI 1.07, 2.43).

**Conclusions:** Low adherence to traditional Chinese postpartum practices was associated with higher EPDS scores indicating PPD symptoms, especially in the domain of housework-related and social activity restrictions. Psychosocial stress and unsatisfactory practical support related to low adherence to postpartum practices might contribute to PPD. Longitudinal study and clinical assessment would be needed to confirm these findings.

## Introduction

The weeks following delivery are a critical period for the long-term health and well-being of the mother and her infant(s) (1). Globally, many cultures observe specific postpartum practices to help the mother recuperate after birth (2). Although postpartum practices vary across cultures, most of them include postnatal care for the mother in a 30–40-day period of rest (3). In Chinese cultures, postpartum practices have been particularly well-documented into a systematic custom known as “doing-the-month” or “confinement” (4). During the 1-month confinement period, Chinese postpartum women are expected to follow many stringent restrictions regarding diet, hygiene, housework-related and social activities, and cold contact (5–7). These traditional restrictions could introduce complex non-uniformed co-occurring but controversial biological, psychological, and socio-environmental changes during postpartum (7–9), of which the health impacts are hard to quantify and have not yet been comprehensively investigated.

Overlapping with the confinement period, postpartum depression (PPD) is one of the most common and disabling but easily unnoticed complications of child-bearing (6, 10), affecting approximately 10–20% of women across different cultural settings (10, 11). Postpartum depression is the major unipolar depressive disorder that occurs in the year after giving birth, with onset mainly within the first 6 weeks postpartum according to International Statistical Classification of Diseases, 10th Revision (ICD-10) (4, 12). Postpartum depression does not only cause maternal morbidity, characterized by episodes of guilt, irritability, exhaustion, anxiety, and sleep disorders (12), but also disrupts downstream infant care and family dynamics (13, 14). Postpartum mental health problems in low- and middle-income countries were suggested to be more severe than those in high-income countries (14, 15). The reported prevalence of PPD in China ranged from 9.4 to 27.4% (16), which could be underestimated due to low public awareness and stigma about mental disorders (17), and could vary according to reporting style (11) or screening timing (18). The identified risk factors of PPD are multifactorial, involving physical, emotional, and social factors (19), suggesting a potentially important role of “doing-the-month” that can fulfill biopsychosocial elements in the etiology or the prevention of PPD (20, 21).

In the Chinese culture, a high adherence to doing-the-month (to follow most traditional postpartum practices) is intended to guarantee practical and social support for the mother, and to promote maternal physical and mental health (4, 22). However, the impact of the adherence to the postpartum practices on PPD remains unknown.

In a systematic review including 16 studies in predominantly Chinese populations (4), eight studies suggested that doing-the-month was associated with a protective effect on PPD (5, 7, 23–28), while four studies indicated the opposite (29–32) and four showed null effect (33–36). The mixed evidence was further complicated by the heterogeneity of “doing-the-month” (6, 22, 34, 37), such as the number, content, and domain of adhered postpartum practices. In most of the previous studies, “doing-the-month” was investigated as a unitary practice, whereas indeed it constitutes practices in diet, hygiene, housework-related and social activities, and cold contact with different adherence levels in the population (5). Existent investigations are inadequate on the health impact of adherence to each specific domain. In addition, primary caregiver (4), parity (38), medical care coverage, and quality (39) may modify the relationship between adherence to postpartum practices and PPD.

Studying the health implications of Chinese postpartum practices might gain valuable insights into prevention or early intervention for mental disorder in the perinatal period in Chinese and other populations. In this cross-sectional study, we aimed to explore the associations between adherence to the traditional Chinese postpartum practices and PPD among postpartum women in Hunan province of China. We hypothesized that adherence to the traditional Chinese postpartum practices is associated with PPD.

## Materials and Methods

### Study Setting and Participants

This is a cross-sectional study conducted in Hunan Province in China between September 2018 to June 2019. The participants were recruited from Hunan Provincial Maternal and Child Health Care Hospital (MCHH) in Changsha and several rural township health centers and urban community health centers in Hunan Province. All potential participants were contacted by researchers when they came for their postnatal appointments (4–10 weeks after delivery), mostly for the routine postpartum examination (around 42 days after delivery) in obstetric clinics. Electronic informed consent was obtained at the enrollment along with an online self-administered questionnaire administered through mobile phones. The women were included if they: (1) were at 4–10 weeks after delivery; (2) owned a cellphone; and (3) could understand and respond to the online questionnaire in Chinese. All participants were invited by trained research staff to complete the online questionnaire on their cellphones after the appointments in the obstetric clinic, with immediate assistance available to potential inquiries during data collection. This study protocol was approved by the ethical review committee of Xiangya School of Public Health Central South University.

Thousand hundred and twenty-six postpartum women were initially invited with 68 excluded due to having birth less than 4 weeks or more than 10 weeks at enrollment, and 3 individuals who provided the same response for all items in the questionnaire were also excluded. The remaining 955 women were included in the study, 632 from MCHH in Changsha and 323 from other municipalities in Hunan Province (Appendix 1 in Supplementary Material).

### Assessment of Adherence to Chinese Postpartum Practices

The exposure of interest was the adherence to Chinese postpartum practices. We adapted a “Practice of Doing-the-month Questionnaire” that has 30 items under four domains (9 under restriction on diet, 14 under restriction on housework-related and social activities, 4 under restrictions on personal hygiene, and 3 under restriction on cold contact) (5). For adherence to each item, participants responded with “yes” or “no.” The content validity index and Cronbach's α of the original questionnaire were 0.95 and 0.86, respectively (5). Questionnaire revision was based on evidence from a pre-testing study in Hunan, without errors from translation (Appendix 2 in Supplementary Material). To minimize missing data, the electronic questionnaire was designed in a “must-enter” format for all items regarding postpartum practices. Additionally, other information regarding postpartum practices was collected at the same time (i.e., location for postpartum practices, primary caregiver, satisfaction with experience of conducting postpartum practices), as well as sociodemographic, obstetric, and psychological characteristics. The satisfaction with experience of conducting postpartum practices was evaluated by a five-level Likert scale, with scores ranging from 1 (least satisfactory) to 5 (most satisfactory).

### Assessment of Postpartum Depression Symptoms

The primary outcome was PPD assessed by the Chinese version of the Edinburgh Postnatal Depression Scale (EPDS) (40, 41). The Chinese EPDS has been validated to show satisfactory specificity and sensitivity for early identification of PPD (40, 41). The 10 items, scored as 0, 1, 2, or 3 to indicate increasing symptoms, explored mood, pleasure, guilt, anxiety, fear, ability to cope, insomnia, sadness, and self-injury. The total score is calculated by summing the scores of each item, with a maximum score of 30. EPDS ≥ 10 was classified as having PPD symptoms in this study, as for the Chinese women the 9/10 threshold performs substantially better than the conventional 12/13 threshold in identifying depression (42). The internal consistency of the EPDS scale, as assessed by Cronbach's α in this study, was 0.89.

### Statistical Analysis

Firth's bias-reduced logistic regression (43) using R package “logistf” (44) was employed to estimate the odds ratio (OR) and 95% confidence interval (CI) of having PPD symptoms according to adherence to postpartum practices. Multivariable linear regression was used to estimate the mean differences in continuous EPDS scores. Adherence was first categorized into “high,” “moderate,” and “low” levels according to the tertile counts of adhered items (yes = 1, no = 0) with a total score of 30. Moreover, this tertile categorization of adherence was applied for satisfaction with the experience of conducting postpartum practices, and four practice domains with a total score of 9, 4, 14, 3 for diet, hygiene, activity, and cold contact, respectively. Furthermore, tertile adherence to every domain was analyzed with and without mutually adjusting for other tertile practice domains, and every single item was analyzed with and without mutually adjusting for items within domains. We conducted trend tests using the Likert scale of satisfaction or the counts of adhered items as a continuous variable. We adjusted for potential confounders including education level, planned pregnancy, family history of PPD, depression diagnosis before pregnancy, primary caregivers, and recruitment locations (MCHH or not). In additional analyses, we adjusted for maternal age, household income level, occupation, parity, the health status of the newborn baby, expected gender of the newborn baby, feeding mode to evaluate influence from these factors. Maternal age extracted from clinic information system had a missing value for 201 participants. Since in total only approximately 2% of participants had missing values on other covariates, we excluded them in the regression analyses. Additionally, we performed stratified analyses to evaluate potential effect measure modification by recruitment locations, parity, primary caregivers. We performed tests of heterogeneity by evaluating the *p*-value of the interaction term between each exposure variable and the potential modifying factors. Furthermore, we performed a cluster analysis on 30 postpartum practices to investigate the relationship between practice patterns and PPD (Appendix 3 in Supplementary Material) using the FactoMineR package (45, 46).

## Results

Table 1 presents the characteristics of the study sample. Overall, 73.4% of the participants had a bachelor or a graduate degree, 54.0% of women have had given birth previously, and 63.5% were planned pregnancies. Only 18 (1.8%) women had a history of depression before pregnancy and 30 (3.1%) reported a family history of PPD. Using predefined 9/10 as the cut-off point of EPDS as afore-mentioned, 36.8% were classified as having PPD symptoms.

**Table 1:** Socio-demographic and obstetric characteristics of postpartum women in Hunan, China, 2018–2019 (*n* = 955).

	**N**	**(%)**
**SOCIO-DEMOGRAPHIC CHARACTERISTICS**		
**Ethnicity**		
Han	926	(97.0)
Others	29	(3.0)
**Residence address**		
City	728	(76.2)
Town	86	(9.0)
Village	141	(14.8)
**Occupation**		
Civil servant	208	(21.8)
Company employee	257	(26.9)
Self-employed people	82	(8.6)
Farmers and peasant workers	51	(5.3)
Others	357	(37.1)
**Education**		
Under senior high	79	(8.3)
Senior high	175	(18.3)
Bachelor	600	(62.8)
Graduate	101	(10.6)
**Monthly household income (kRMB)**		
<5	167	(17.5)
5–10	324	(33.9)
10–15	207	(21.7)
15–20	116	(12.1)
>20	141	(14.8)
**OBSTETRIC CHARACTERISTICS**		
**Planned pregnancy**	606	(63.5)
**First pregnancy**	439	(46.0)
**Gender of baby consistent with expectation**	709	(74.2)
**Delivery mode**		
Vaginal delivery	576	(60.3)
Cesarean delivery	379	(39.7)
**Infant feeding**		
Exclusive breast-feeding	572	(59.9)
Exclusive formula-feeding	49	(5.1)
Mixed feeding	334	(35.0)

Overall, participants had various satisfactory levels toward the experience of conducting postpartum practices. Over 70% of participants reported a medium or higher rating (≥3) regarding the experience of conducting postpartum practices, whereas <20% of participants gave the highest rating (5/5) (Table 2, Supplementary Table 1).

**Table 2:** Odds Ratio (OR) for PPD symptoms (EPDS scores ≥ 10) according to postpartum practice adherence^a^.

	**EPDS scores ≥10 vs**. ** <10**				
**Postpartum practices**	**N**	**Model1^b^ OR**	**(95% CI)**	**Model2^c^ OR**	**(95% CI)**
**Satisfaction with experience^d^**					
High rating (4–5)	377	1.00	(Reference)	/	
Not bad (3)	317	1.40	(0.95, 2.05)	/	
Low rating (1–2)	240	2.25	(1.40, 3.63)	/	
**Overall adherence^e^**					
High adherence (18–30)	294	1.00	(Reference)	/	
Moderate adherence (14–17)	306	1.11	(0.74, 1.68)	/	
Low adherence (0–13)	356	1.26	(0.82, 1.94)	/	
**Restriction on diet**					
High adherence (7–9)	239	1.00	(Reference)	1.00	(Reference)
Moderate adherence (5–6)	353	0.99	(0.63, 1.54)	1.05	(0.65, 1.68)
Low adherence (0–4)	363	0.98	(0.62, 1.53)	0.95	(0.58, 1.56)
**Restriction on housework-related and social activity**					
High adherence (9–14)	313	1.00	(Reference)	1.00	(Reference)
Moderate adherence (7–8)	253	1.16	(0.75, 1.78)	1.24	(0.80, 1.94)
Low adherence (0–6)	389	1.61	(1.07, 2.43)	1.81	(1.13, 2.90)
**Restriction on personal hygiene**					
High adherence (2–4)	207	1.00	(Reference)	1.00	(Reference)
Moderate adherence (1)	247	1.04	(0.62, 1.73)	0.89	(0.52, 1.51)
Low adherence (0)	501	0.79	(0.50, 1.23)	0.65	(0.39, 1.08)
**Restriction on cold contact**					
High adherence (3)	442	1.00	(Reference)	1.00	(Reference)
Moderate adherence (2)	284	1.04	(0.70, 1.54)	1.01	(0.66, 1.53)
Low adherence (0–1)	229	1.13	(0.73, 1.74)	1.01	(0.61, 1.67)

a *Binary logistic regression was used to estimate odds of PPD symptoms (EPDS scores ≥10) compared with EPDS Scores <10*.

b *Model1 adjusted for education level (under bachelor, bachelor/graduate), planned pregnancy (yes, no), family history of postpartum depression (yes, no), depression diagnosis before pregnancy (yes, no), primary caregiver during 1-month postpartum (own mother, mother-in-law, husband/self, all others), recruitment location (Hunan provincial maternal and child health care hospital in Changsha, any other clinic)*.

c *Model2 additionally mutually adjusted other practice domains based on Model1*.

d *Scores ranged from 1 (least satisfactory) to 5 (most satisfactory)*.

e *Adherence was determined by number of completed items*.

Table 2 shows the associations between PPD symptoms and the satisfactory rating or adherence to traditional Chinese postpartum practices. A lower rating on postpartum practice experience was associated with higher odds of PPD symptoms (*P* for trend <0.001). The OR estimates were 1.11 and 1.26 for moderate and low adherence groups, respectively, though the CIs included null values.

Among the four domains in postpartum practices, low adherence to housework-related and social activity restrictions was associated with symptoms of PPD compared with the high adherence group (Table 2). The OR for low adherence to activity restrictions was 1.61 (95% CI 1.07, 2.43) and the *P* for trend was 0.061, without adjusting for other domains. The suggestive trends in associations for decreasing restrictions on housework-related and social activity were strengthened in the model mutually adjusting for other domains simultaneously (*P* for trend 0.022). While no apparent associations were found for having PPD symptoms and the restrictions on diet, personal hygiene, or cold contact.

Analyses for the mean difference in continuous EPDS scores showed similar findings with binary PPD symptoms in both overall and domain adherence, where both moderate and low adherence to postpartum practices appeared to be associated with higher EPDS scores (adjusted difference 1.07, 95% CI 0.20, 1.94 for overall moderate adherence; and adjusted difference 1.72, 95% CI 0.84, 2.60 for overall low adherence, *P* for trend <0.001) (Table 3).

**Table 3:** Mean difference in EPDS scores according to postpartum practice experience and adherence^a^.

	**Difference in EPDS scores**			
**Postpartum practices**	**Model1^b^**	**(95% CI)**	**Model2^c^**	**(95% CI)**
**Satisfaction with experience^d^**				
High rating (4–5)	0.00	(Reference)	/	
Not bad (3)	2.07	(1.28, 2.86)	/	
Low rating (1–2)	4.35	(3.49, 5.21)	/	
**Overall adherence^e^**				
High adherence (18–30)	0.00	(Reference)	/	
Moderate adherence (14–17)	1.07	(0.20, 1.94)	/	
Low adherence (0–13)	1.72	(0.84, 2.60)	/	
**Restriction on diet**				
High adherence (7–9)	0.00	(Reference)	0.00	(Reference)
Moderate adherence (5–6)	0.17	(−0.78, 1.12)	0.15	(−0.81, 1.12)
Low adherence (0–4)	0.17	(−0.78, 1.12)	−0.41	(−1.42, 0.59)
**Restriction on housework-related and social activity**				
High adherence (9–14)	0.00	(Reference)	0.00	(Reference)
Moderate adherence (7–8)	1.02	(0.10, 1.94)	1.29	(0.35, 2.24)
Low adherence (0–6)	2.95	(2.11, 3.78)	3.43	(2.48, 4.39)
**Restriction on personal hygiene**				
High adherence (2–4)	0.00	(Reference)	0.00	(Reference)
Moderate adherence (1)	0.73	(−0.31, 1.76)	−0.17	(−1.22, 0.87)
Low adherence (0)	−0.33	(−1.25, 0.59)	−1.37	(−2.38, −0.37)
**Restriction on cold contact**				
High adherence (3)	0.00	(Reference)	0.00	(Reference)
Moderate adherence (2)	0.37	(−0.46, 1.21)	0.04	(−0.89, 0.81)
Low adherence (0–1)	0.99	(0.09, 1.88)	0.04	(−0.96, 1.04)

a *Multiple linear regression was used to estimate the mean difference in EPDS scores*.

b *Model1 adjusted for education level (under bachelor, bachelor/graduate), planned pregnancy (yes, no), family history of postpartum depression (yes, no), depression diagnosis before pregnancy (yes, no), primary caregiver during 1-month postpartum (own mother, mother-in-law, husband/self, all others), recruitment location (Hunan provincial maternal and child health care hospital in Changsha, any other clinic)*.

c *Model2 additionally mutually adjusted other practice domains based on Model1*.

d *Scores ranged from 1 (least satisfactory) to 5 (most satisfactory)*.

e *Adherence was determined by number of completed items*.

The numbers of women reporting adherence were varied across postpartum practices, generally with the lowest adherence to restrictions on personal hygiene, while the highest adherence in avoiding sexual intercourse (Supplementary Table 2). Analyses for each practice item showed adherence to many housework-related and social activity restrictions were independently associated with lower EPDS scores. For diet, personal hygiene, and cold contact constraint, there were no consistent patterns found for single items. Some associations were observed for single items, but the results were inconsistent after accounting for other items within the category.

No apparent effect modifications were observed for recruitment location, parity, primary caregiver in associations between postpartum practices and PPD symptoms (Supplementary Table 3).

**Figure 1:**
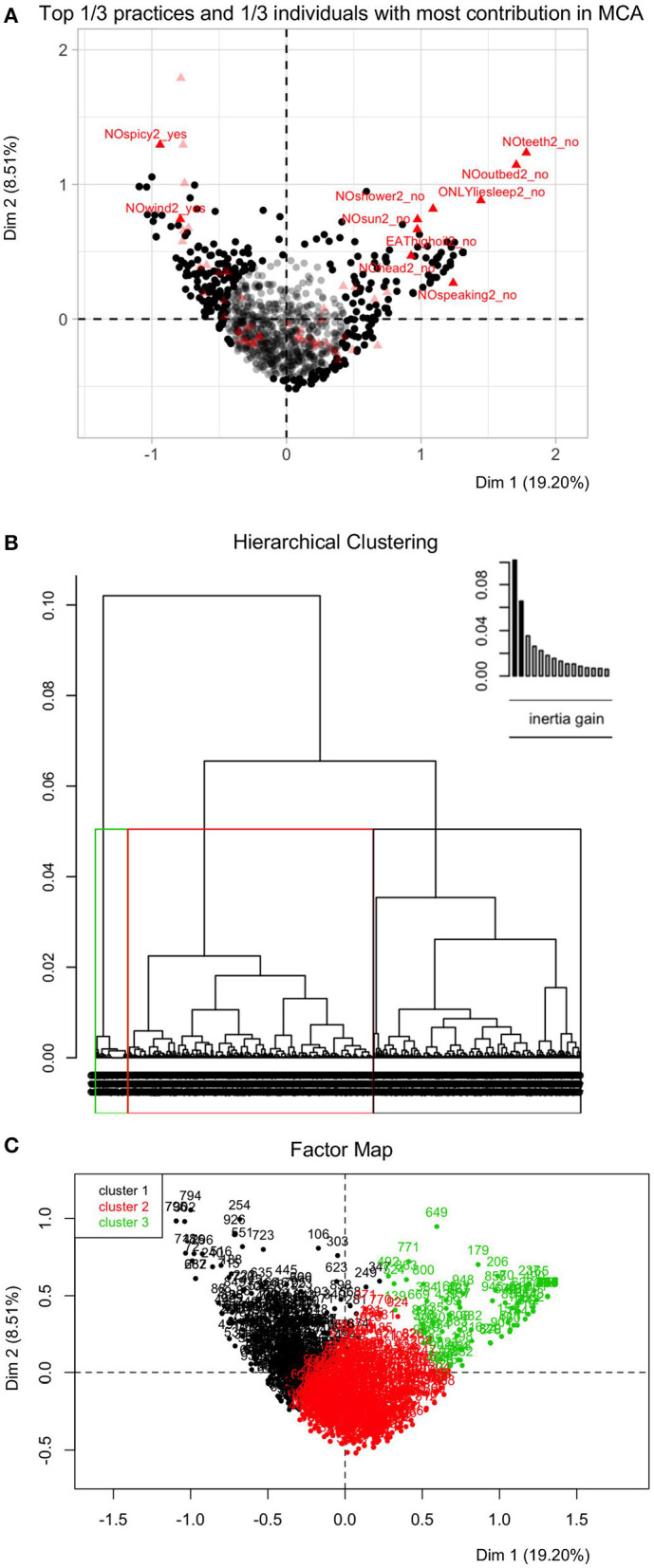
Hierarchical clustering on principle components (HCPC) for postpartum practices among postpartum women in Hunan, China, 2018–2019 (*n* = 955). **(A)** Top 1/3 practices and top 1/3 individuals with most contribution in multiple correspondence analysis (MCA). MCA retains information from the first converted 19 dimensions (principal components) in this study, which accounts for 80% of information of the original 30 postpartum practices. The first two dimensions in MCA accounts for 19.20 and 8.51% information, respectively. On the plane formed by the first two dimensions, the dark black points denote 318 out of 955 individuals with most contribution in MCA results. The bright red triangles denote 10 out of 30 postpartum practices with most contribution in MCA results, including avoiding spicy and “hot” food, not brushing teeth, avoiding getting out of bed etc. **(B)** Dendrograms of hierarchical clustering on 19 MCA dimensions. Bar plots above dendrograms explain the gain of within-cluster inertia for each dendrogram, which assist in determining the levels at which the hierarchical tree was cut for clustering. The number of clusters were determined when no significant difference in between-/within-cluster inertia could be detected after adding/subtracting one more cluster. **(C)** Clusters are shown on two-dimension MCA factor map: Cluster 1 (343/955) denotes cluster with few items adhered, Cluster 2 (516/955) denotes cluster with mixed items adhered, and Cluster 3 (96/955) denotes cluster with most items adhered. There are no units for this type of graph since the principal component has no units.

Three clusters were identified based on adherence to each postpartum practice, which may reflect the degree of adherence to these items (Figure 1). Specifically, Cluster 1 (343/955) was characterized as following very few practices. In contrast, Cluster 3 (96/955) conducted almost all practice items in each domain. Cluster 2 (516/955), the largest cluster, had a mixed adherence to postpartum practices. Regression analysis using the cluster variable as a predictor suggested low adherence to postpartum practices was associated with having PPD symptoms compared with the mixed adherence, while the cluster with almost all items completed did not show a strong association (Table 4, Supplementary Table 4).

**Table 4:** Mean difference in EPDS scores according to postpartum practice cluster patterns^a^.

	**Difference in EPDS scores**	
**Postpartum practice cluster**	**Model1^b^**	**(95% CI)**
Cluster 3 (most items adhered)	0.15	(−1.11, 1.42)
Cluster 2 (mixed items adhered)	0.00	(Reference)
Cluster 1 (few items adhered)	1.72	(0.95, 2.48)

a *Multiple linear regression was used to estimate the mean difference in EPDS scores*.

b *Model1 adjusted for education level (under bachelor, bachelor/graduate), planned pregnancy (yes, no), family history of postpartum depression (yes, no), depression diagnosis before pregnancy (yes, no), primary caregiver during 1-month postpartum (own mother, mother-in-law, husband/self, all others), recruitment location (Hunan provincial maternal and child health care hospital in Changsha, any other clinic)*.

Supplementary Table 5 shows numbers of overall adhered items and within every practice domain in each cluster that differed from each other (*P* < 0.001). Consistently, Cluster 1 (very few practices adhered) was over-represented by having PPD symptoms, also linked to the least satisfactory level of “doing-the-month” experience (1 out of 5 scores).

In addition, the results did not markedly change in models further adjusted for more social-demographic and obstetric characteristics in sensitivity analyses. Finally, our findings did not change when accounting for maternal age when using a smaller sample size (*n* = 754).

## Discussion

In this study, we evaluated the associations between adherence to the traditional Chinese postpartum practices (“doing-the-month”) and depressive symptoms in women during 4–10 weeks postpartum in Hunan, China. We found that low and moderate adherence to postpartum practices, as well as lower satisfactory ratings on “doing-the-month” experience, was associated with PPD. This impact was particularly consistent in the domain of housework-related and social activity restrictions. Cluster analyses confirmed the findings where women following very few practices were over-represented by having PPD symptoms and dissatisfaction toward the experience of “doing-the-month.”

Previous studies on “doing-the-month” have addressed both the health benefits or detriments of the Chinese postpartum practices, with inconsistent conclusions on mental effect (4, 37, 38). One major issue is that the naive exposure classification failed to capture the diverse domains of postpartum practices and overlooked the biopsychosocial functioning of practice patterns. Among little evidence considering the heterogeneity of postpartum practices, the estimated impact on PPD is also controversial. One study involving 202 Taiwanese women at 4–6 weeks after birth found that every 1-score increment out of the total 108 adherence scores to postpartum practices decreased the odds of PPD by 0.97, after adjusting education, parity, infant feeding mode, location for “doing-the-month” (5). By contrast, another study in Wuhan, Hubei province using the same version of questionnaire showed a crude positive association between adherence to “doing-the-month” and PPD at 6 weeks (22). These findings show that the differences in study populations (e.g., sample size, parity, socio-economic class), in outcome measurement (e.g., screening questionnaires used and the timing), and in confounding control might also lead to mixed findings.

Briefly, the association of low adherence to postpartum practices with PPD in the current study might be explained by the biopsychosocial model of illness development (20, 21) and the causal pie model of outcome occurrence (47, 48). For instance, interactions of certain factors under biological, psychological and social domains could form sufficient causal mechanisms and trigger PPD occurrence. Our findings might reflect mechanisms including biopsychosocial changes such as elevated psychosocial stress and unsatisfactory social support associated with postpartum practices during a heightened sensitive period for depression occurrence. However, these mechanisms would require confirmations in longitudinal research. Indeed, our study has shown a mismatch between expectation and reality: most women would conduct traditional postpartum practices, but less than 20% expressed complete satisfaction toward their own “doing-the-month” experiences. A study in Beijing, China reported 55% (178/327) perceived “doing-the-month” useful (38), which is not an overwhelming victory as well. Moreover, women who considered “doing-the-month” as unhelpful showed twice the odds of PPD (38, 49). A Taiwanese study found complete dissatisfaction with instrumental support was very strongly positively correlated with EPDS scores in postpartum women, and women with a greater level of social support displayed fewer depressive symptoms (34, 50). The findings strengthen the conjecture that the demand for quality postpartum care is still unmet, and inadequate support may influence completion of postpartum practices, leading to stress or even depression (34, 39).

On one hand, low-adherence-related psychosocial stress may affect PPD development. Sources of stress include financial stress (51), family conflicts, and concerns on against “doing-the-month” as a social norm (39, 52). In other words, failed expectations in conducting interested practices may raise a concern of future health or add tension to the family relationship (22, 53), also refusion toward traditions may cause psychological stress and family conflicts (7, 38). For example, forced low adherence to the activity domain may be linked with the stress of returning to work for a living (51, 54) or insufficient maternal and paternity leave (55). A most recent prospective cohort study in Shanghai, China reported that women who left their homes during the first month postpartum could have at least 90% higher risks of PPD compared with those who never went outside (15). Additionally, traditional Chinese postpartum practices generally do not encourage exercises/workouts in this period (7), but limiting housework and exercises may have a different impact on mental status. Evidence-based guidelines are warranted to fill the blanks in traditional Chinese postpartum practices (56), and to confront disagreement on postpartum exercises.

On the other hand, postpartum practices possibly contain protective elements to postpartum physical and mental relief (5, 6, 57). Satisfactory practical support could be an important element. Housework-related and social activity restrictions mainly represented the possible protective role of “doing-the-month” on preventing PPD in our study. For example, a study among 341 Taiwan women reported squatting as a predictor of high anxiety and depression scores in 2015 (58). This suggests that practical support on works requiring long-time standing/squatting or heavy lifting for postpartum women may offer some protective efforts. Notably, how women mentally perceive housework (54) or even how they evaluate received support may also affect their mental status (38, 49). In most families, practical support usually includes a caregiver(s) to free the mother from domestic housework duties (38). As our study shows more than 70% of primary caregivers were family members, satisfactory practical support from families might also reinforce family bonds and benefit mental health. Thus, limiting housework and social activities might provide considerable rest for physical recovery and mental wellness (4, 59). A prospective cohort study found that women who slept <6 h per night were twice more likely to suffer from PPD compared with those who slept 8 h (15). In this way, satisfactory practical support to new mothers after birth can be critical to maintaining subsequent self-esteem and wellness (4, 22).

In addition, mother-in-law as a caregiver used to be identified as a risk factor for PPD (4), conversely for own mother as a key helper (34). But there is no strong evidence of effect modification of primary care-givers in associations between postpartum practices and PPD in our study. The content and quality of postpartum support should receive more attention. To overcome disagreement from inter-generational beliefs and cultural taboos about postpartum activities, it is suggested to prepare a care team and detailed postpartum care plans beforehand, including housework distribution (1). As nearly half of postpartum women were following guidance from parents or parents-in-law, future health education on postpartum recovery should consider the family as a whole (39). This may serve to reduce psychosocial stress and improve practical support for the prevention of PPD.

For the relatively inconsistent evidence in restrictions on personal hygiene and cold contact, more replications are needed to explore their associations with PPD. The health belief model (60, 61) might be helpful to explain the degrees of adherence to postpartum practices, and the related mental health effects. Briefly, future investigations on adherence to postpartum practices might also want to evaluate how women perceive the susceptibility to complications of childbirth, the health benefits of these restrictions together with the received support, the barriers to adherence (52, 54).

### Strengths

Briefly, our study not only investigated overall adherence to “doing-the-month” or several behavioral items, but also analyzed postpartum practices by comprehensive domains and items. We found adherence to traditional postpartum practices differed by domain and by item, along with their relationships with PPD. This heterogeneity related to adherence indicates that “doing-the-month” remains a common but unstandardized custom (7, 8). Thus, via analyses of overall adherence and further break-down, on the one hand, our findings could probably capture the consistent relationship between adherence to traditional postpartum practices and PPD. On the other hand, as postpartum practices are observed globally, and western cultures generally have women navigate the postpartum transition independently (1, 2), this Chinese study might provide an informative reference for prevention or early intervention for mental disorder in the perinatal period in Chinese and other populations. Moreover, our study had a large sample size (~1,000 participants) and controlled for many important confounders in multiple regression, while some of the limited studies about adherence to postpartum practices and PPD (~200 participants) used correlation analyses (22). Finally, to our best of knowledge, this study is one of the few studies investigating the impact of housework-related restrictions on postpartum mental health (54).

### Limitations

Nevertheless, this cross-sectional study has several limitations. First, postpartum women were only contacted after 1-month confinement, and less healthy individuals were less likely to be enrolled or to finish the questionnaires. Secondly, most of the participants were sampled from the provincial hospital with higher education and income level than average, which might limit the external validity of the findings. But the recruitment location was adjusted and did not show significant effect modification. In addition, the modified version of PDQ has not been validated in our study. However, since our revision was based on feedback from the participants in our pilot survey, it is less likely to threaten the validity. Also, we did not obtain information on life events stress in the perinatal period which might be a risk factor of PPD. But one of the most common sources of perinatal stress is financial stress, and the results where we additionally adjusted for household income did not significantly change. Additionally, there was no clinical assessment to evaluate biological changes and physical conditions including hormone levels or sleep quality other than mental health screening. Finally, reverse causality and confounding cannot be ruled out as explanations, as depressive symptoms which began before childbirth might account for a portion of PPD identified, and could act as a confounding in the practice-PPD pathway and could in turn alter postpartum physical and social activeness. But first, we adjusted family history of PPD and diagnosis of depression before pregnancy, second, the screening time in our study was just about the typical onset timing of PPD [symptoms begin within 6 weeks postpartum in 80% of cases] (6). Future studies may employ repeated assessments at shorter intervals throughout the perinatal period to identify onset timing of depressive symptoms (62), also for early prevention.

## Conclusions

In conclusion, “doing-the-month” remains a common but heterogeneous custom for postpartum women. Overall low adherence to the traditional Chinese postpartum practices was associated with higher EPDS scores indicating PPD symptoms, especially in the domain of housework-related and social activity restrictions. Psychosocial stress and unsatisfactory practical support related to low adherence to postpartum practices might contribute to PPD. The protective elements within Chinese postpartum practices might gain valuable insights into prevention or early intervention for mental disorder in the perinatal period in Chinese and other populations. Longitudinal study and clinical assessment of PPD would be needed to further explore the health impacts of adherence to multifaceted domains of postpartum practices considering the biopsychosocial approach.

## Data Availability Statement

The raw data supporting the conclusions of this article will be made available by the authors, without undue reservation.

## Ethics Statement

The studies involving human participants were reviewed and approved by The ethical review committee of Xiangya School of Public Health Central South University. The patients/participants provided their written informed consent to participate in this study.

## Author Contributions

PG: data analysis, methodology, writing original draft, reviewing, and editing. DX: conceptualization, methodology, writing original draft, reviewing, and editing. ZL: conceptualization, methodology, data analysis, reviewing, and editing. HH: conceptualization, methodology, and data analysis. PB and BT: conceptualization, reviewing, and editing. CZ and XJ: data curation, investigation, methodology, and project administration. WG: conceptualization, funding acquisition, investigation, methodology, project administration, resources, supervision, reviewing, and editing. All authors contributed to the article and approved the submitted version.

## Conflict of Interest

The authors declare that the research was conducted in the absence of any commercial or financial relationships that could be construed as a potential conflict of interest.

## Publisher's Note

All claims expressed in this article are solely those of the authors and do not necessarily represent those of their affiliated organizations, or those of the publisher, the editors and the reviewers. Any product that may be evaluated in this article, or claim that may be made by its manufacturer, is not guaranteed or endorsed by the publisher.
