# Supplementary material for: Adherence to Traditional Chinese Postpartum Practices and Postpartum Depression: A Cross-Sectional Study in Hunan, China
Source: Front Psychiatry. 2021 Jul 27;12:649972. doi: 10.3389/fpsyt.2021.649972 (PMC8353075; doi:10.3389/fpsyt.2021.649972)
Supplement: Supplementary file 1 [file Data_Sheet_1.docx]

Supplementary Material

**Table S1. Attitude and characteristics of conducting postpartum practices among postpartum women^a^.**

|  | No. | (%) |
| --- | --- | --- |
| Satisfaction with experience of conducting postpartum practices ^b^ |  |  |
| 1 - least satisfactory | 96 | (10.3) |
| 2 | 144 | (15.4) |
| 3 | 317 | (33.9) |
| 4 | 220 | (23.6) |
| 5 - most satisfactory | 157 | (16.8) |
| Location for postpartum practices |  |  |
| Own home | 506 | (54.2) |
| Parents' home | 123 | (13.2) |
| Parent-in-law's home | 221 | (23.7) |
| Postpartum care center/others | 84 | (9.0) |
| Primary caregivers during confinement |  |  |
| Own mother | 341 | (36.5) |
| Mother-in-law | 247 | (26.4) |
| Husband/self | 112 | (12.0) |
| Postpartum care center/hired personnel/others | 234 | (25.0) |

^a^ 21 participants’ responses out of 955 were missing. ^b^ Scores ranged from 1 (least satisfactory) to 5 (most satisfactory).

**Table S2. Mean difference in EPDS scores according to single postpartum practice adherence^a^.**

|  | Difference in EPDS scores | | | | | | |
| --- | --- | --- | --- | --- | --- | --- | --- |
| Postpartum practices | N | Model1 **^b^** | (95% | CI) | Model2 **^c^** | (95% | CI) |
| **Restriction on diet** |  |  |  |  |  |  |  |
| Consumed chicken/fish/meat soup | 532 | 0.83 | (0.11, | 1.54) | 1.01 | (0.24, | 1.79) |
| Ate high-protein food | 387 | 0.14 | (-0.59, | 0.87) | -0.07 | (-0.88, | 0.74) |
| Ate high-fat food | 197 | 0.51 | (-0.38, | 1.40) | 0.53 | (-0.45, | 1.50) |
| Drank sweet wine or brown sugar water | 398 | 0.29 | (-0.45, | 1.02) | 0.12 | (-0.68, | 0.91) |
| Avoided raw food, cold food or ice products | 653 | -0.71 | (-1.48, | 0.06) | -0.69 | (-1.49, | 0.11) |
| Avoided legumes | 295 | -0.63 | (-1.42, | 0.16) | -0.73 | (-1.56, | 0.10) |
| Avoided spicy and ‘hot’ food | 870 | -0.40 | (-1.67, | 0.88) | 0.29 | (-1.14, | 1.71) |
| Take soft food only | 753 | -0.36 | (-1.24, | 0.52) | -0.07 | (-1.02, | 0.89) |
| Did not eat salty food | 828 | -1.15 | (-2.20, | -0.10) | -1.12 | (-2.29, | 0.05) |
| **Restriction on housework-related and social activity** | | | | | | | |
| Avoided squatting (i.e., showering the baby) | 506 | -1.36 | (-2.08, | -0.64) | -0.21 | (-0.99, | 0.57) |
| Avoided standing for long periods | 608 | -1.48 | (-2.22, | -0.75) | -0.46 | (-1.25, | 0.33) |
| Avoided carrying heavy objects | 859 | -2.00 | (-3.21, | -0.80) | -0.72 | (-1.92, | 0.49) |
| Avoided getting out of bed | 101 | 0.55 | (-0.63, | 1.72) | 1.62 | (0.29, | 2.94) |
| Avoided exposure to sunshine | 189 | 0.03 | (-0.88, | 0.94) | 0.01 | (-0.97, | 0.99) |
| Avoided exposure to wind/draughts | 751 | -0.73 | (-1.60, | 0.15) | 0.77 | (-0.13, | 1.68) |
| Avoided reading, watching TV, or sewing | 389 | -2.09 | (-2.84, | -1.34) | -0.79 | (-1.63, | 0.04) |
| Remained lying down | 117 | -0.29 | (-1.41, | 0.83) | 0.25 | (-0.95, | 1.45) |
| Did not go out especially not visit others’ homes | 610 | -0.50 | (-1.25, | 0.25) | -0.16 | (-0.91, | 0.59) |
| Avoided talking excessively | 167 | -1.51 | (-2.48, | -0.54) | -0.78 | (-1.85, | 0.28) |
| Did not have sexual intercourse | 937 | -0.79 | (-3.40, | 1.81) | 0.31 | (-2.24, | 2.86) |
| Did not go to temples and burn incense | 774 | -1.06 | (-1.96, | -0.15) | -0.78 | (-1.66, | 0.11) |
| Avoided visitors entering postpartum woman’s room | 385 | -1.06 | (-1.79, | -0.33) | 0.20 | (-0.55, | 0.96) |
| Kept a good mood (avoided sadness) | 550 | -3.78 | (-4.48, | -3.07) | -3.41 | (-4.20, | -2.62) |
| **Restriction on personal hygiene** |  |  |  |  |  |  |  |
| Did not wash hair | 238 | -0.15 | (-0.97, | 0.68) | -0.79 | (-1.84, | 0.26) |
| Did not take a bath | 179 | 0.69 | (-0.23, | 1.61) | 1.39 | (0.14, | 2.64) |
| Did not brush teeth | 97 | 0.03 | (-1.18, | 1.24) | -0.48 | (-1.93, | 0.97) |
| Used only boiled water when needed | 310 | 0.12 | (-0.67, | 0.90) | 0.10 | (-0.72, | 0.92) |
| **Restriction on cold contact** |  |  |  |  |  |  |  |
| Avoided contact with cold water | 777 | -1.06 | (-1.99, | -0.14) | -0.99 | (-0.80, | 0.25) |
| Wore a hat | 562 | -0.31 | (-1.04, | 0.41) | -0.02 | (-1.57, | 1.43) |
| Avoided exposing the skin | 729 | -0.79 | (-1.63, | 0.05) | -0.66 | (-0.26, | 1.55) |

^a^ Multiple linear regression was used to estimate the mean difference in EPDS scores. ^b^ Model1 adjusted for education level (under bachelor, bachelor/graduate), planned pregnancy (yes, no), family history of postpartum depression (yes, no), depression diagnosis before pregnancy (yes, no), primary care giver during one-month postpartum (own mother, mother-in-law, husband/self, all others), recruitment location (Hunan provincial maternal and child health care hospital in Changsha, any other clinic). ^c^ Model2 additionally mutually adjusted other practices within domains based on Model1.

**Table S3. Stratified analysis of odds Ratio (OR) for PPD symptoms (EPDS scores >=10)^a^.**

|  | MCHH (n=632) | | | Any other clinic (n=323) | | |  | First Pregnancy (n=439) | | | Multiple parities (n=516) | | |  |
| --- | --- | --- | --- | --- | --- | --- | --- | --- | --- | --- | --- | --- | --- | --- |
| Postpartum practices | OR^b^ | (95% | CI) | OR^b^ | (95% | CI) | P ^c^ | OR^d^ | (95% | CI) | OR^d^ | (95% | CI) | P ^c^ |
| **Overall adherence** |  |  |  |  |  |  | 0.942 |  |  |  |  |  |  | >0.999 |
| High adherence (18-30) | 1.00 | (Reference) | | 1.00 | (Reference) | |  | 1.00 | (Reference) | | 1.00 | (Reference) | |  |
| Moderate adherence (14-17) | 1.18 | (0.70, | 1.99) | 1.07 | (0.51, | 2.24) |  | 1.12 | (0.57, | 2.17) | 1.10 | (0.66, | 1.85) |  |
| Low adherence (0-13) | 1.28 | (0.75, | 2.19) | 1.32 | (0.59, | 2.97) |  | 1.22 | (0.63, | 2.35) | 1.22 | (0.70, | 2.14) |  |

^a^ Binary logistic regression was used to estimate odds of PPD symptoms (EPDS scores >=10) compared with EPDS Scores <10. Adjusted for education level (under bachelor, bachelor/graduate), planned pregnancy (yes, no), family history of postpartum depression (yes, no), depression diagnosis before pregnancy (yes, no), primary care giver during one-month postpartum (own mother, mother-in-law, husband/self, all others). ^c^ P value for heterogeneity (P value for the interaction term). ^d^ Adjusted for education level (under bachelor, bachelor/graduate), planned pregnancy (yes, no), family history of postpartum depression (yes, no), depression diagnosis before pregnancy (yes, no), primary care giver during one-month postpartum (own mother, mother-in-law, husband/self, all others), recruitment location (Hunan provincial maternal and child health care hospital in Changsha, any other clinic).

**Table S4. Odds Ratio (OR) for PPD symptoms (EPDS scores >=10) according to postpartum practice cluster patterns^a^.**

|  | EPDS Scores >=10 vs <10 | | | |
| --- | --- | --- | --- | --- |
| Postpartum practice cluster | N | OR **^b^** | (95% | CI) |
| Cluster 3 (most items adhered) | 96 | 1.14 | (0.84, | 1.59) |
| Cluster 2 (mixed items adhered) | 516 | 1.00 | (Reference) | |
| Cluster 1 (few items adhered) | 343 | 1.35 | (1.12, | 1.68) |

^a^ Binary logistic regression was used to estimate odds of PPD symptoms (EPDS scores >=10) compared with EPDS Scores <10. ^b^ Model1 adjusted for education level (under bachelor, bachelor/graduate), planned pregnancy (yes, no), family history of postpartum depression (yes, no), depression diagnosis before pregnancy (yes, no), primary care giver during one-month postpartum (own mother, mother-in-law, husband/self, all others), recruitment location (Hunan provincial maternal and child health care hospital in Changsha, any other clinic).

**Table S5. Description of the clusters for 30 postpartum practices and supplementary variables^a^.**

|  | Few  items adhered  (n=343) | Mixed  items adhered (n=516) | Most  items adhered  (n=96) |
| --- | --- | --- | --- |
| Active variables: Practices to follow | 11 (9,12) ^b^ | 17 (15,19) | 26 (23,29) |
| **Restriction on diet** | 4 (3,5) | 5 (4,6) | 9 (7,9) |
| Ate high-protein food | Under* ^c^ | / | Over** |
| Ate high-fat food | Under* | Under | Over** |
| Avoided raw food, cold food or ice products | Under** | Over** | Over* |
| Drank sweet wine or brown sugar water | Under | / | Over* |
| Avoided legumes | Under* | / | Over** |
| Consumed chicken/fish/meat soup | Under | / | Over* |
| Avoided spicy and ‘hot’ food | Under* | Over* | / |
| Did not eat salty food | Under* | Over* | Over |
| Take soft food only | Under** | Over* | Over |
| **Restriction on housework-related and social activity** | 5 (4,6) | 8 (7,9) | 12 (10,14) |
| Did not go out especially not visit others’ homes | Under* | Over | Over* |
| Avoided getting out of bed | Under* | Under* | Over** |
| Avoided exposure to sunshine | Under | Under* | Over** |
| Avoided talking excessively | Under* | / | Over** |
| Avoided exposure to wind/draughts | Under** | Over** | Over |
| Did not have sexual intercourse | Under | Over | / |
| Did not go to temples and burn incense | Under | / | Over |
| Avoided squatting (i.e., showering the baby) | Under** | Over** | Over |
| Avoided standing for long periods | Under** | Over** | Over* |
| Avoided crying/getting upset | Under** | / | Over* |
| Avoided visitors entering ‘doing-the-month’ room | Under* | Over* | Over* |
| Avoided reading, watching TV, or sewing | Under** | Over* | Over** |
| Avoided carrying heavy objects | Under** | Over* | / |
| Remained lying down | Under* | Under* | Over** |
| **Restriction on personal hygiene** | 0 (0,1) | 1 (0,1) | 3.5 (3,4) |
| Did not wash hair | Under* | / | / |
| Did not take a bath | Under* | Under | Over** |
| Did not brush teeth | Under* | Under | Over** |
| Used only boiled water when needed | Under* | / | Over** |
| **Restriction on cold contact** | 1 (1,2) | 3 (2,3) | 3 (3,3) |
| Avoided contact with cold water | Under** | Over* | Over |
| Wore a hat | Under** | Over** | Over* |
| Avoided exposing the skin | Under** | Over** | Over |
| Supplementary variables |  |  |  |
| Residence address | City | / | Village* |
| Occupation | / | / | Farmers*; Migrant workers |
| Education | Bachelor | / | Under senior high; Senior high |
| Monthly household Income (kRMB) | 15-20 | / | < 5* |
| Planned Pregnancy | No | / | Yes |
| Gender of baby consistent with expectation | Inconsistent | Consistent | / |
| Education | Bachelor | / | Under senior high; Senior high |
| EPDS score>=10 | Yes | No | / |
| Satisfaction with experience of conducting postpartum practices ^e^ | 1* | 5* | / |
| Location for postpartum practices | / | / | Parents-in-law's home |
| Primary caregivers during confinement | / | / | Mother-in-law |

^a^ The hypergeometric test is considered significant if the p-value is less than 0.05. Test statistics is v-test value not showing here. Variable categories significantly linked to the cluster where values emphasized by the asterisks (***) denote a v-test value of ≥20, (**) a v-test value of 20–10, (*) a v-test value of 10–5 and no asterisk a v-test value of 5–0.

^b^ Median (inter-quartile range) of number of adhered items.

^c^ ‘Over’ refers to ‘over-representation’; ‘Under’ refers to ‘Under-representation’. ‘/’ refers to ‘not statistically significant’ in the hypergeometric test.

^d^ In the Chinese culture, many people believe that good adherence to postpartum practices can avoid some health problems in older age.

^e^ Scores ranged from 1 (least satisfactory) to 5 (most satisfactory).

**Appendix 1. The geographic distribution of the included participants**

The remaining 955 women were included into the study, 632 (66.2%) from MCHH in Changsha and 323 (33.8%) from other municipalities in Hunan Province [125 (13.1%) from Yiyang; 66 (6.9%) from Chenzhou; 47 (4.9%) from Zhuzhou; 22 (2.3%) from Xiangtan; 19 (2.0%) from Huaihua and 44 (4.6%) others].

**Appendix 2. The process of modifying ‘Practice of Doing-the-month Questionnaire’**

We adapted a ‘Practice of Doing-the-month Questionnaire’ (PDQ) developed and validated by Chien to collect specific information on the practice of ‘doing-the-month’ (Chien et al., 2006): deleting several items ( ‘avoided ‘toxic’ food’, ‘did not drink plain water’, and ‘ingested Eucommia ulmoides’) as our pre-testing found that those items were either difficult to understand or rarely practiced in Hunan, and adding a few new items (‘avoided exposure to sunshine’, ‘drank rice wine or brown sugar water’, ‘did not brush teeth’, ‘avoided exposing the skin’, ‘Avoided getting out of bed’, ‘avoided talking excessively’, and ‘avoided legumes’) per the feedback from the pre-testing participants. We also combined ‘avoided eating ‘cold’ food’ and ‘avoided eating raw food’ into one item ‘avoided eating cold and/or raw food’ as in the local parlance those two were always discussed together. In the end, we developed the modified PDQ with 30 items under four domains (9 under dietary adjustment, 14 under physical and social activities, 4 under personal hygiene, 3 under warmth retention).

**Appendix 3. Hierarchical clustering on the principal components selected by Multiple Correspondence Analysis**

We first used multiple correspondence analysis (MCA) to denoise the dataset for stable clustering. Then we performed hierarchical clustering on principle components (HCPC) to group our participants into distinct clusters with similar practice patterns (Husson 2010). Apart from using cluster indicator in regression, hypergeometric test was used to identify over- or under-represention of other variables from the questionnaire across clusters. MCA and HCPC functions were carried out in R environment with FactoMineR package (Husson 2015; R Development Core Team 2008).

Rationale of determining number of clusters in hierarchical clustering: A division of *Q* clusters is strongly suggested if the increase of between-cluster difference between *Q-1* and *Q* clusters is significantly greater than the one between *Q* and *Q-1* clusters. In other words, we chose *Q* to minimize this ratio *R* (let the increase of between-cluster difference when moving from *Q-1* to *Q* clusters):

$$R=\frac{\Delta Q}{\Delta(Q+1)}$$
